# A Preliminary Feasibility Study on Hemodynamic Changes Following Feyh-Kastenbauer Retractor Insertion During Transoral Robotic Surgeries

**DOI:** 10.7759/cureus.38804

**Published:** 2023-05-09

**Authors:** Nitika Goel, Sonali Kaushal, Deepanshu Dhiman, Naresh K Panda, Tanvir Samra

**Affiliations:** 1 Anaesthesia and Intensive Care, Postgraduate Institute of Medical Education and Research, Chandigarh, IND; 2 Anaesthesia, Dr. Yashwant Singh (YS) Parmar Medical College and Hospital, Nahan, IND; 3 Otolaryngology, Postgraduate Institute of Medical Education and Research, Chandigarh, IND

**Keywords:** sevoflurane, fentanyl, hemodynamic changes, transoral robotic surgery, feyh-kastenbauer retractor

## Abstract

Introduction

Transoral robotic surgery (TORS) has become increasingly popular for the removal of pharyngeal and laryngeal cancers with the objective to improve functional and aesthetic outcomes. Feyh-Kastenbauer (FK) retractor is one such routinely used retractor during TORS. The setting up of this retractor has been seen to be accompanied by hemodynamic fluctuations.

Methodology

This prospective observational study was carried out on 30 patients undergoing TORS. All patients were administered general anesthesia using a pre-defined anesthesia protocol. The primary outcome was to compare hemodynamic fluctuations following endotracheal intubation with that after FK retractor insertion. Any requirement of a bolus dose of sevoflurane and fentanyl was recorded in response to hemodynamic fluctuations recorded in secondary outcomes.

Results

There was no statistically significant increase in mean heart rate, systolic, diastolic, and mean arterial blood pressure from baseline to endotracheal intubation and following retractor insertion (p=0.810, p=0.2, p=0.6, p=0.3 respectively). On subgroup analysis, hypertensive patients reported a greater rise in blood pressure following two minutes post FK retractor insertion compared to non-hypertensive patients (p=0.03). Out of 30 patients, five patients required a bolus dose of sevoflurane.

Conclusion

FK retractor insertion had a comparable hemodynamic response as endotracheal intubation during TORS. Hypertensive patients showed a rise in blood pressure at both endotracheal intubations and at FK retractor insertion.

## Introduction

Transoral robotic surgery (TORS) has become increasingly popular for the removal of pharyngeal and laryngeal cancers with the objective to improve functional and aesthetic outcomes without worsening survival. The presence of long working arms provides the greatest advantage in reaching the posteriormost part of the oral cavity. The main advantage is allowing better surgical access, via the mouth, to areas where hands and instruments probably reach with difficulty through the mouth itself [1]. TORS is a relatively new approach to removing cancers from areas of difficult access like the throat, base of the tongue, and low down in the bottom of the tonsil [1].

Unfortunately, without TORS, the surgery required can be quite invasive, including splitting the mandible (mandibulotomy) to expose the base of the tongue. Presently partial laryngectomies are also being performed completely through the mouth with the robot technique. However, having read about all these advantages, TORS is also associated with some potential sequelae. TORS procedure requires the placement of complex retractors followed by the docking of robotic arms inside the oral cavity [2]. Feyh-Kastenbauer (FK) retractor (Gyrus Medical Inc, Tuttlingen, Germany) is one such routinely used retractor during TORS. It allows better exposure of the oropharynx using a longer tongue blade. The precise placement and setting up of robotic arms inside the oral cavity requires a lot of manipulation of retractors and robotic instruments. The setting up of these instruments has been seen to be accompanied by hemodynamic fluctuations which were found to be comparable to those following endotracheal intubation. Through this study, we propose to compare the hemodynamic fluctuations occurring following endotracheal intubation and FK retractor insertion. The study hypothesized that FK retractor insertion is associated with a significant increase in blood pressure as compared to following endotracheal intubation.

## Materials and methods

Methodology

**Figure 1:**
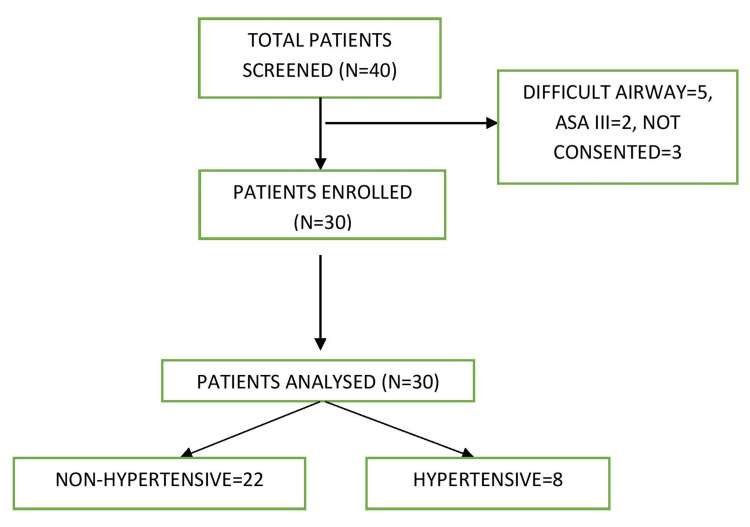
Consolidated Standards of Reporting Trials (CONSORT) flow diagram ASA = American Society of Anaesthesiology

The present study was conducted in the Department of Anaesthesia and Intensive Care in collaboration with the Department of Otolaryngology-Head Neck surgery at a tertiary care center in Northern India. The present study is a prospective observational study. The study was approved by the institutional ethics committee of Postgraduate Institute of Medical Education and Research (PGIMER) with letter no INT/IEC/2020/JPL-52 and was also registered with the central clinical trials registry (CTRI) with reference no. CTRI/2020/07/026857. A total of 30 patients were enrolled in the study after receiving written informed consent (Figure 1).

The inclusion criteria were: Patients undergoing TORS for oral cavity pathologies, American Society of Anaesthesiology (ASA) category I and II, and aged 18-60 years. Patients with suspected difficult airways were excluded. Patients undergoing robotic-assisted ENT surgeries for other than oral cavity cancers and ASA category III and above were also excluded.

All patients were assessed one day prior to surgery and a detailed pre-anaesthetic check-up was done including pre-operative orders given by a trained anesthetist. The following relevant points were noted from this pre-anaesthetic check-up: history of smoking, snoring, the parameter of complete airway examination: mouth opening (MO), Mallampati grading (MPG), thyromental distance (TMD), sternomental distance (SMD) and dentition. Within the operation theatre, all ASA essential monitoring was placed including non-invasive blood pressure (NIBP), pulse oximetry (SpO2), electrocardiogram (ECG) and end-tidal CO2 (EtCO2) monitoring. Baseline vitals including heart rate (HR), systolic blood pressure (SBP), diastolic blood pressure (DBP), mean arterial pressure (MAP), and oxygen saturation by pulse oximetry were noted. The intravenous line was secured. A standard induction protocol for general anesthesia was followed which included injection fentanyl 1 µg/kg, injection propofol 2-2.5 mg/kg, and injection atracurium 0.5 mg/kg. This was followed by endotracheal intubation after complete muscle relaxation checked by loss of jaw tone and after bag-mask ventilation for four minutes following the administration of muscle relaxation. Following the confirmation of the endotracheal tube, the tube was secured and the inhalation agent sevoflurane was titrated to maintain minimum alveolar concentration (MAC) between 1.2-1.4. The maintenance of anesthesia was done by nitrous 60%, oxygen 40%, and sevoflurane titrated to achieve a MAC of 1.2. Ten minutes following the stabilization of MAC at 1.2, the patient was handed over to the surgeons for the insertion of the FK retractor. Hemodynamic parameters which included HR, SBP, DBP, and MAP were recorded continuously at one-minute intervals following endotracheal intubation and thereafter at retractor insertion till the end of the surgical procedure. Other parameters which were recorded included EtCO2, MAC, end-tidal concentration of inhalation agent, SpO2, and requirement of a bolus dose of opioid. The time of retractor insertion and removal and the total duration of retractor placement were also recorded.

Hypertension was defined as an SBP of more than 120% of the baseline value or more than 160 mmHg, whereas hypotension was defined as an SBP of less than 70% of the baseline value or less than 90 mmHg. Tachycardia and bradycardia were defined as HR greater than 120 beats/min and less than 60 beats/min, respectively. The episodes of hypertension, hypotension, tachycardia, and bradycardia were recorded throughout the study. A dysrhythmia was defined as any ventricular or supraventricular premature beat or any sustained rhythm other than sinus. The incidence of dysrhythmia after intubation and following retractor insertion was recorded.

If SBP or DBP is more than 120% of baseline value or more than 160 mmHg for SBP and 100 mmHg for DBP, a bolus dose of inhalation agent-sevoflurane was delivered to increase the MAC to 1.8%. Similarly, a rise in heart rate greater than 120 beats/min was dealt with with a bolus dose of fentanyl 0.5 µg/kg.

Outcomes

Primary outcome was to compare the hemodynamic fluctuations i.e the mean change from baseline in the heart rate, systolic blood pressure, diastolic blood pressure, and mean arterial blood pressure following endotracheal intubation with that following FK retractor insertion during TORS.

Secondary outcome was intervention done to decrease the hypertensive response observed during intubation and retractor insertion. The intervention is defined as a bolus dose of an opioid drug or increasing the depth of anesthesia measured in terms of the end-tidal concentration of the inhalation agent and MAC.

Statistical analysis

Data entry and analysis were done with the help of Office Excel 2007 (Microsoft, Redmond, WA, USA) and SPSS software version 21 (IBM Corp., Armonk, NY, USA). Descriptive analysis was done using frequency, percentages, and mean (+/-S.D.). The normality of data was checked by the Kolmogorov-Smirnoff test. Statistical analyses of the data for each hemodynamic parameter were performed by an appropriate test of significance i.e. t-test and two-way analysis of variance with repeated measures (rANOVA). p<0.05 was considered statistically significant.

## Results

A total of 40 patients were screened from which five patients were excluded due to anticipated difficult airway, three patients did not give consent and two patients were excluded being the ASA III category. The data of 30 patients who underwent TORS was collected and a final analysis was done. The demographic details of the patients including age, gender, height, weight, BMI, and ASA category are summarized in Table 1. The variables of the demographic data were normally distributed throughout the study population.

**Table 1:** Demographic details Data represented as * mean with standard deviation, # absolute number (%), MO- Mouth opening, MPG- Mallampati Grading, TMD- Thyromental distance, SMD- Sternomental distance and FB- Finger breadth

Demographic Details of the Patients	
Age(years)^*^	55.35 ± 14.52.
Gender^#^	
Male	27 (91.7%)
Female	3 (8.3%)
Height (cm)^*^	162.09 ± 7.38
Weight(kg)*	61.88 ± 15.62.
BMI (kg/m^2^)*	23.48 ± 5.48
ASA I	10 (30%)
ASA II	20 (70%)
ASA III	-
History of smoking	17 (54.2%)
Non-smoker^#^	13 (45.8%)
History of Snoring	7 (16.7%)
No snoring	23 (83.3%)
MO^#^(>3FB)	30 (100.0%)
MPG^#^II	20 (70.8%)
MPG III	10 (29.2%)
TMD^#^(> 3FB)	30 (100.0%)
SMD^#^ (> 12 cm )	30 (100.0%)
Dentition^#^(normal/ loose teeth/ edentulous)	20 (83.3%)
Loose teeth	6 (4.2%)
Edentulous	4 (1.25%)

Primary outcomes

Heart Rate (HR)

**Figure 2:**
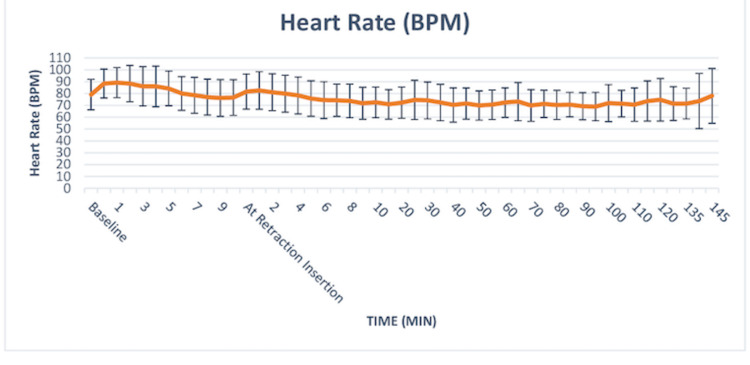
The change in heart rate over time was not statistically significant following endotracheal intubation or FK retractor insertion (Friedman Test: χ2 = 39.3, p = 0.810)

The mean heart rate at baseline was 79.04 ± 12.89 at intubation was 88.38 ± 12.19, at one-minute post-intubation was 89.29 ± 12.72 and at 10 minutes was 76.58 ± 15.09. At retractor insertion, the HR was 81.58 ± 14.79, at one-minute post retractor insertion was 82.50 ± 15.88, and at 10 minutes post retractor insertion was 72.52 ± 12.91. The change in heart rate over time was not statistically significant following endotracheal intubation or FK retractor insertion (Friedman Test: χ2=39.3, p=0.810). A post-adhoc analysis was also done to see at which specific time points the heart rate differed significantly. The heart rate (BPM) differed significantly from the baseline at the following time points: 135 minutes post retractor insertion (RI) (p=0.018), 140 minutes post RI (p=0.01), and 145 minutes post RI (p=0.01). These time points correlated to the time of extubation of the patient. Although the maximum change from the baseline timepoint was observed at the one-minute post-intubation timepoint none of the patients had HR > 120 (tachycardia) or HR < 60 beats per minute (bradycardia) (Figure 2).

Systolic Blood Pressure

The mean SBP at baseline was 129.25 ± 21.67mmHg which further showed an increase to more than 140 mm of Hg at one-minute post-intubation (142.88 ± 30.73). Following this, a rise in blood pressure was noted one minute post retractor insertion, where the mean SBP was 140.38 ± 27.3. Though there was a rise in mean SBP following intubation and retractor insertion, it was less than 120% of baseline and statistically not significant (p=0.2). Further, during the surgical period the change in mean BP was not significant either clinically or statistically. A post hoc analysis was performed and values at three-time points i.e. 135 minutes, 140, and 145 minutes post retractor insertion were statistically significant. These were time points of extubation and recovery from where such hemodynamic changes can be observed.

Diastolic Blood Pressure

The diastolic blood pressure followed a similar trend as the systolic blood pressure did, with a rise in blood pressure over baseline at one-minute post-intubation and one-minute post-retractor insertion. The baseline mean DBP was 78±11.85, at one-minute post-intubation was 86.79 ± 16.59 and at post one-minute retractor insertion was 84.17 ±15.49. These variations in diastolic blood pressure also failed to reach any clinical or statistical significance(p-0.6). The DBP did not show a large deviation from the baseline means DBP at the rest of the time points. The data for DBP was normally distributed hence parametric test and the repeated measures of the ANOVA test were used for statistical inference.

Mean Arterial Blood Pressure (MAP)

**Figure 3:**
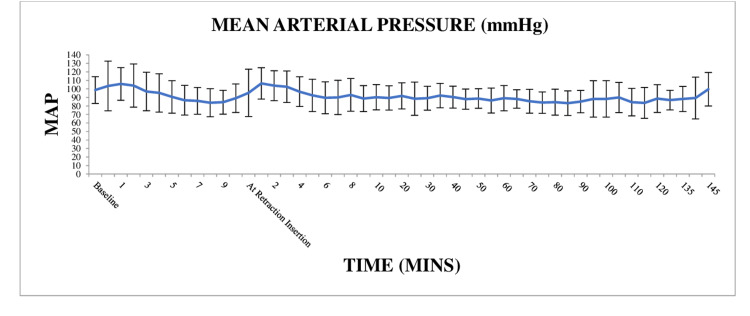
The variation in the MAP was not statistically significant following endotracheal intubation or FK retractor insertion (p=0.3) MAP = mean arterial pressure, FK = Feyh-Kastenbauer

Clinical variations were observed in the MAP over time, and changes were observed at the time of intubation and at retractor insertion as seen in both the systolic and diastolic blood pressure measurements. These changes were not found to be significant clinically or statistically and did not require any intervention (p=0.3). The post hoc analysis revealed only two significant values, that is at 140 and 145 minutes post retractor insertion corresponding to the time of extubation and recovery when systolic and diastolic blood pressures were also significantly reduced from the baseline. The variation in the MAP is depicted in Figure 3.

Secondary outcomes

Out of 30 patients, five patients required the administration of a bolus dose of sevoflurane to increase the MAC to 1.6 following retractor insertion. None of the patients required additional doses of fentanyl. However, no statistically significant increase in MAC was observed throughout the surgery. A rise is seen from a baseline value (starting from the time when delivery of inhalation was initiated) to the time of intubation where maximum end-tidal sevoflurane concentration was noted as required during induction. Further, during the maintenance phase the MAC was maintained between 1-1.2 with no significant variations in the delivery of the inhalation agent even during retractor insertion and following it. The mean MAC increased from a minimum of 0.36 at the baseline timepoint to a maximum of 1.18 at the four-minute post RI timepoint and then decreased to 1.00 at the 145-minute post RI timepoint. This change was also not statistically significant (p=0.188). On post hoc analysis none of the values both for end-tidal sevoflurane and MAC had significant differences from baseline versus intubation and at retractor insertion. Statistically, significant values were found after 125 minutes of retractor insertion till 145 minutes (end of the procedure). This corresponds to the time when surgery was completed and patients were in the recovery phase of general anesthesia. 

Hemodynamic Changes in Hypertensive Patients

An intragroup analysis comparing hypertensive versus non-hypertensive patients was done, in which out of 30 patients, eight patients were hypertensive. In the hypertensive group, the mean SBP just before intubation (108.62 ±39.10 mm of Hg) was significantly lower compared to the non-hypertensive group (147.88 ±36.51 mm of Hg, p=0.02). Following retractor insertion at two minutes, the increase in systolic blood pressure was clinically more in hypertensive patients compared to non-hypertensive patients. We also found a significant increase in DBP and MAP at two minutes post retractor insertion (p=0.03) on comparison of hypertensive and non-hypertensive patients. This required administration of a bolus dose of sevoflurane to increase MAC to 1.6 in five hypertensive patients two minutes after retractor insertion. Further, during points of retractor manipulation and removal, the hypertensive group showed a significant increase in heart rate from 20 minutes post RI to up to 50 minutes post retractor insertion (Table 2).

**Table 2:** Comparison of heart rate in both the groups and various time points

Heart Rate (BPM)	Hypertension		P value for comparison of the two groups at each of the timepoints (Wilcoxon-Mann-Whitney Test)
	Present	Absent	
	Mean (SD)	Mean (SD)	
20 Minutes Post RI	81.00 (11.78)	68.20 (12.09)	0.041
25 Minutes Post RI	85.00 (13.24)	69.80 (16.01)	0.034
30 Minutes Post RI	83.86 (11.77)	69.36 (15.44)	0.052
35 Minutes Post RI	83.00 (14.94)	66.77 (12.81)	0.039
40 Minutes Post RI	81.17 (9.13)	65.38 (13.92)	0.018
45 Minutes Post RI	81.50 (8.12)	66.92 (12.53)	0.012
50 Minutes Post RI	78.33 (10.07)	65.67 (11.34)	0.024

These hemodynamic fluctuations up to 50 minutes post retractor insertion all indicate increased sympathetic stimulation in the hypertensive group to retractor insertion in comparison to non-hypertensive patients. There were no statistically significant differences in MAC, EtCO2, and end-tidal sevoflurane concentrations.

Though all patients successfully underwent surgery and were extubated at the end, one patient had to be re-explored due to bleeding at the surgical site. Another patient experienced delayed extubation, had unequal pupil size, and had to be ventilated electively in the Intensive care unit for 24 hours, following which the patient recovered and was extubated. The non-contrast computed tomography of the patient reported normal. Both these patients were hypertensive and required bolus doses of inhalational agents to maintain the SBP within 120% of baseline.

## Discussion

As the primary outcome of interest, we observed comparable hemodynamic responses following endotracheal intubation and FK retractor insertion. We also found an exaggerated hypertensive response to both endotracheal intubation and FK retractor insertion in the hypertensive subgroup. There was also an increased requirement for inhalational agents in the hypertensive group as compared to the normotensive group.

The adverse cardiovascular changes and catecholamine discharge seen during laryngoscopy and tracheal intubation appear in two phases. The effects of laryngoscopy should be distinguished from the effects seen while the endotracheal tube is placed through the trachea. Shribman et al. showed the differences between these two events. Even with stable anesthesia, laryngoscopy alone without intubation can cause a supraglottic stimulus. As a result, SBP and DBP increase in contrast to the measurements before induction. However, no significant increase in HR occurs during laryngoscopy. An increase in BP is due to norepinephrine, while an increase in HR is due to epinephrine discharge. Infraglottic stimulus caused by placing the endotracheal tube occurs in phase two. In this situation, an extra cardiovascular response and catecholamine discharge occur. Stress response increases at this stage and both SBP and DBP measurements increase by 36-40% in contrast to control levels. HR levels increase more than 20% with tracheal intubation in contrast to laryngoscopy [3,4]. Kings et al. in the year 1951 first elucidated this response to intubation and laryngoscopy [5]. This is a reflex cardiovascular response with afferent pathway-mediated vagus which occurs due to stimulation of receptors in the base of the tongue and on lifting the epiglottis. This hemodynamic response may not have implications in young ASA category I patients but it needs to be adequately controlled in old patients and higher ASA category where it may have serious implications.

FK retractor comprises a tongue blade and a suspension arm which helps fix the retractor to the left side of the operation table. Once adequately placed inside the oral cavity, it allows better exposure to the surgical field and movement of robotic arms. The precise placement and setting up of robotic arms inside the oral cavity requires a lot of manipulation of retractors and robotic instruments. Nevertheless, these manipulations are also associated with significant hemodynamic fluctuations which are found equivalent to those occurring following endotracheal intubation. This study compares the hemodynamic changes following endotracheal intubation with FK retractor insertion during TORS.

The primary aim of our anesthetic management is to maintain the hemodynamics of the patient within a particular range and avoid large variations within the same. Advances in anesthesia in the availability of drugs and various aids for intubation have largely helped us in reducing this hemodynamic response to laryngoscopy and intubation. These methods include deepening the plane of anesthesia [6], the use of different laryngoscopy blades and different types of laryngoscopies [7-10], and the use of various drugs before any airway manipulations [10-15]. Multiple drugs in combination and alone have been used. These include anesthetic agents, opioids, beta-blockers, alpha-agonist like clonidine and dexmedetomidine, gabapentin, lignocaine infusions, and magnesium sulfate.

In our study, the hemodynamic response to laryngoscopy and intubation has been studied for the first 10 minutes. After these 10 minutes, the FK retractor was inserted by the otorhinolaryngologist (Figures 2, 3). We chose 10 minutes as the interval between intubation and FK retractor insertion as maximum sympathetic stimulation following laryngoscopy and intubation occurs within 10 minutes only and subsides thereafter [16]. There were no significant changes in hemodynamic parameters at various timepoint of intubation, and retraction insertion as well as there was no increase in the requirement of MAC or opioids in most of the patients. In the intragroup analysis of hypertensive patients versus non-hypertensive patients, we found significantly lower blood pressure in the hypertensive group compared to non-hypertensive patients, just before intubation. This can be explained by the additive effects of anti-hypertensive agents along with the anesthetic agents, leading to an exaggerated fall in blood pressure. Post intubation we did not any statically significant increase in SBP, though DBP and MAP showed a significant increase. This can be explained by the enhanced release of catecholamines and increased sensitivity of peripheral blood vessels resulting in increased vasoconstriction and raised DBP and MAP. There was also a statistically significant increase in HR 20 mins following retractor insertion which persisted till 60 mins following retractor insertion in hypertensive patients. Throughout this time it persisted less than 120 beats/min and did not require any additional dose of fentanyl. 

Jeyarajah et al. in their review article on perioperative concerns in patients with robotic-assisted ENT surgeries advocate the use of opioids like fentanyl, remifentanil, and beta-blockers to obtund the sustained hemodynamic response to retractor placement [17]. In another recent case series of four cases, they described the use of fentanyl boluses and beta-blockers to reduce the sympathetic response. While the literature search shows a scarcity of data on the sympathetic response occurring following FK retractor insertion during TORS, there is no study that directly compares this response with endotracheal intubation. The findings of the present study indicate that if induction of anesthesia, the plane of anesthesia, and the initial opioid dose are given adequately the hemodynamic responses following endotracheal intubation and FK retractor insertion are quite similar. Additional interventions in the form of an increase in anesthetic concentration were required in hypertensive patients following retraction insertion. The sympathetic response to the surgical gag insertion lasted for up to 60 minutes after the retractor insertion in these patients as seen by the hemodynamic alteration. Therefore, it is important to have a heightened level of vigilance in hypertensive patients to attenuate their hemodynamic responses. Though statistically not significant, we observed more complications like bleeding, delayed extubation, and transient increase in intracranial pressure resulting in unequal pupils.

Limitation

As this is only a pilot study, due to the small sample size it is not powered enough for assessing the primary outcome. However, our study did answer the pragmatics of recruitment and the feasibility of a larger study. Second, we did not quantify and compare the amount of fentanyl used in hypertensive and non-hypertensive patients. Therefore, we feel more future research on the role of opioids in attenuating the hemodynamic response following FK retractor insertion can be planned.

## Conclusions

FK retractor insertion had a comparable hemodynamic response to endotracheal intubation during TORS. Hypertensive patients showed a rise in blood pressure at both endotracheal intubations and at FK retractor insertion requiring increased requirement of anesthetic agents.
